# Significado de la comodidad para pacientes crónicos hospitalizados

**DOI:** 10.15649/cuidarte.1915

**Published:** 2021-08-20

**Authors:** Yadira Cardozo García, Ángela María Salazar Maya, Carmen Liliana Escobar Ciro

**Affiliations:** 1 . Universidad de Antioquia, Medellín- Colombia. Email: yadira.cardozo@udea.edu.co Autor de correspondencia. Universidad de Antioquia Universidad de Antioquia Medellín Colombia yadira.cardozo@udea.edu.co; 2 . Universidad de Antioquia, Medellín- Colombia. Email: angela.salazar@udea.edu.co Universidad de Antioquia Universidad de Antioquia Medellín Colombia angela.salazar@udea.edu.co; 3 . Universidad de Antioquia, Medellín- Colombia. Email: carmen.escobar@udea.edu.co Universidad de Antioquia Universidad de Antioquia Medellín Colombia Email: carmen.escobar@udea.edu.co

**Keywords:** Comodidad del Paciente, Enfermedad Crónica, Hospitalización., Patient Comfort, Chronic Disease, Hospitalization., Conforto do Paciente, Doença Crônica, Hospitalização.

## Abstract

**Introducción:**

la comodidad componente de calidad de vida, busca la conservación y recuperación de la salud. El objetivo fue comprender el significado de la comodidad del paciente con enfermedad crónica hospitalizado en Medellín-Colombia.

**Material y métodos:**

investigación etnográfica particularista, con 14 participantes, a través de entrevistas semiestructuradas y observación. Análisis se realizó con herramientas de la teoría fundamentada, se dio lectura y relectura, codificación de datos, y se generaron categorías/subcategorías hasta la saturación teórica a través del muestreo teórico.

**Resultados:**

categorías: Interactuando con otros: El compañero del lado y La muerte de otro. Interactuando con el equipo de salud: Atributos de la atención; y Entre la comodidad e incomodidad con los que atienden. Elementos: comunicación e información.

**Discusión:**

la comodidad está dada en parte por la interacción que los pacientes tienen con el equipo de salud, en especial con enfermería. Igual se comprendió lo que generan comodidad e incomodidad en los pacientes. Los atributos personales como la empatía, la competencia profesional y el uso de un lenguaje adecuado son los más nombrados. Igualmente, la información y la comunicación son vitales en la percepción de comodidad.

**Conclusiones:**

el significado que los pacientes le atribuyen a la comodidad está dada por la interacción con el equipo de salud, sus atributos personales e información que se brinda sobre su estado y evolución de salud como de los efectos de su tratamiento. Entre los que causan incomodidad: no ser escuchados, la actitud negativa del personal de enfermería, no atender su llamado oportunamente y presenciar la muerte del compañero.

## Abstract (en)

**Introduction:**

comfort, a quality of life component, seeks the preservation and recovery of health.

**Objective:**

was to understand the meaning of comfort in patients with chronic disease hospitalized in Medellin-Colombia.

**Material and methods:**

particularistic ethnographic research, with 14 participants, through semi-structured interviews and observation. Analysis was carried out with grounded theory tools, reading and rereading, data coding, and categories/ subcategories were generated until theoretical saturation through theoretical sampling.

**Results:**

Categories: Interacting with others: The partner on the side and The death of another. Interacting with the health care team: Attributes of care; and Between comfort and discomfort with caregivers. Elements: communication and information.

**Discussion:**

comfort is given in part by the interaction that patients have with the health care team, especially with nursing. It was also understood what generates comfort and discomfort in patients. Personal attributes such as empathy, professional skills and the use of appropriate language are the most frequently mentioned. Likewise, information and communication are vital in the perception of comfort.

**Conclusions:**

the meaning that patients attribute to comfort is given by the interaction with the healthcare team, their personal attributes and the information provided about their health status and evolution as well as the effects of their treatment. Among those that cause discomfort: not being listened to, the negative attitude of the nursing staff, not attending to their call in a timely manner and witnessing the death of a partner.

## Resumo

**Introdução:**

o conforto, um componente da qualidade de vida, busca a preservação e a recuperação da saúde

**O objetivo:**

era compreender o significado do conforto em pacientes com doenças crônicas hospitalizados em Medellín-Colômbia.

**Material e métodos:**

pesquisa etnográfica particularista, com 14 participantes, mediante entrevistas semiestruturadas e observação. A análise foi realizada com ferramentas da teoria fundamentada, leitura e releitura, codificação de dados, e categorias/subcategorias foram geradas até a saturação teórica através de amostragem teórica.

**Resultados:**

Categorias: Interagindo com outros: O parceiro ao lado e A morte de outro; Interagindo com a equipe de saúde: Atributos do cuidado; e Entre conforto e desconforto com os cuidadores. Elementos: comunicação e informação.

**Discussão:**

o conforto é dado em parte pela interação que os pacientes têm com a equipe de saúde, especialmente com a enfermagem. Também foi entendido o que gera conforto e desconforto nos pacientes. Atributos pessoais como empatia, competência profissional e o uso de linguagem apropriada são os mais mencionados. Da mesma forma, a informação e a comunicação são vitais na percepção do conforto. Conclusões: O significado que os pacientes atribuem ao conforto é dado pela interação com a equipe de saúde, seus atributos pessoais e as informações fornecidas sobre o estado de saúde e evolução, assim como os efeitos de seu tratamento. Entre aqueles que causam desconforto: não ser escutados, a atitude negativa do pessoal de enfermagem, não atender prontamente seu chamado e testemunhar a morte do colega.

## Introducción

Las enfermedades crónicas son enfermedades de progresión lenta y larga duración1 en Colombia las enfermedades crónicas y algunos factores de riesgo asociados con estilos de vida son causantes de enfermedad y muerte, rebasando las causadas por violencia y accidentes. Entre ellas están las enfermedades del sistema circulatorio, principalmente la enfermedad isquémica del corazón, la enfermedad cerebro vascular y la hipertensión arterial y sus complicaciones, ocupan el primer lugar, seguido de tumores, enfermedades pulmonares crónicas y enfermedades degenerativas osteoarticulares(2). Igualmente, en Medellín las principales causas de mortalidad en el periodo 2016-2019 son las enfermedades isquémicas del corazón(3).

Como dice Ardila “Este panorama no mejorará mientras nuestro sistema de salud continúe centrado en el manejo de la enfermedad y no en la salud, y las entidades prestadoras de servicios de salud no reconozcan que es más rentable “prevenir que curar””(2). Estas situaciones llevan a reingresos hospitalarios por descompensación o exacerbación de las patologías y las personas ingresan a servicios de hospitalización para el tratamiento de sus enfermedades crónicas y a su vez la hospitalización brinda innumerables experiencias entre las que se encuentran las relacionadas con la comodidad.

La comodidad en la práctica de enfermería ha tenido como finalidad la conservación y recuperación de la salud(4). Además, es considerada un componente importante de la calidad de vida de las personas y muchas de las intervenciones realizadas por las enfermeras, se centran en promover la comodidad(5), y lograr bienestar de la persona cuidada, no solo desde lo técnico o instrumental, sino más allá, en esa búsqueda de atender las necesidades de dicha persona en sus dimensiones, psicológicas, sociales y espirituales en un cuidado holístico.

Muchas autoras se han ocupado de este concepto como Janice Morse quien relata que debería ser el paradigma central para la enfermería(6)-(8); Kolcaba genero la teoría del confort que se ha utilizado en diferentes contextos de cuidado(7).

La comodidad es un concepto multidimensional, que incluye aspectos físicos tanto de las instalaciones(9) como las propias del paciente, y aspectos subjetivos que se relacionan con la sensación de seguridad, confianza, competencia, control personal, sentirse cuidado, valorado, seguro y a gusto(10)

Durante la estancia hospitalaria se generan alteraciones en el confort relacionadas directa o indirectamente con la enfermedad y la hospitalización; que desencadena estrés, que se extiende a los miembros de la familia(11); por tanto, este disconfort traspasa la dimensión física, y afecta el aspecto psicoespiritual, ambiental y social.

Así mismo, la comodidad hace parte de la percepción de satisfacción del paciente, que interesa a la hora de medir la calidad de cuidado brindado por enfermería y la comodidad hace parte de esta percepción o experiencia del paciente(10); sin embargo este concepto se relaciona con otros como bienestar y la calidad de vida. Los tres, son conceptos relacionados que comparten atributos comunes(12).

Koehn, afirma que la teoría de la comodidad contribuye a guiar a las enfermeras en la enseñanza y la aplicación de medidas de confort para mitigar los efectos de molestias o disconfort en muchas situaciones(13), como lo evidencian los estudios de aplicación de la teoría de mediano rango en pacientes terminales, psiquiátricos, pediátricos, con cáncer, quirúrgicos y en trabajo de parto.

Araujo et al., evaluaron la comodidad en cuidadores familiares de pacientes con cáncer y encontraron que esta se relacionaba con la edad y la ocupación del cuidador; los aspectos positivos fueron sentirse amado, la comodidad física del paciente y de su ambiente y la espiritualidad del cuidador. La falta de comodidad se relacionaba con la falta de trabajo remunerado o descanso y concluyeron que el identificar las necesidades de los cuidadores permiten a los profesionales de salud hacer intervención de estas(14). Poveda DC, determinó la validez y la confiabilidad del cuestionario sobre comodidad, en personas hospitalizadas con enfermedad crónica, concluyó que no es un instrumento confiable para su empleo en la población colombiana con enfermedad crónica.

Aunque las autoras parten de la construcción del concepto de comodidad como la satisfacción de las necesidades físicas, psicoespirituales, ambientales y sociales que experimentan los pacientes garantizando los tres tipos de confort: alivio, tranquilidad y trascendencia, como lo propone Katherine Kolcaba(7). Surge la necesidad de revisar la pertinencia cultural del concepto “comodidad” desde la perspectiva de los pacientes hospitalizados por enfermedad crónica previo a su empleo en poblaciones de características similares(15). Por esto, cuando en el quehacer diario de enfermería se habla de comodidad el propósito de este estudio es estudiarlo a mayor profundidad en nuestro contexto colombiano no sólo para fortalecer el cuerpo de conocimientos y la disciplina profesional; sino porque el fin último de la Enfermería es el cuidado el cual busca la comodidad de la persona para así lograr el bienestar. De ahí que explorar el concepto de comodidad en relación con su correspondencia cultural es un reto que enfermería debe asumir, en especial en el cuidado del paciente con enfermedad crónica. Por lo que para la investigación se plantea como pregunta ¿Cuál es el significado de la comodidad del paciente con enfermedad crónica hospitalizado?

## Materiales y Métodos

Se realizó una investigación cualitativa(16) desde una perspectiva etnográfica particularista, la cual se centra en un grupo de personas que tienen algo en común(17), para el caso los pacientes con enfermedad crónica (EC) hospitalizados en los servicios de medicina interna de una institución de salud, de cuarto nivel de atención, del área metropolitana de la ciudad de Medellín, entre febrero de 2018 y octubre de 2019.

Entre los criterios de inclusión se encuentran: adultos mayores de edad, hospitalizados por padecer enfermedad crónica.

Después de tener el aval del Comité de investigación de la institución, se estableció contacto con las enfermeras del área administrativa a las cuales se les presentó el proyecto, y actuaron como porteras(18) para permitir el acceso y acercamiento a los servicios de medicina interna, dos de las investigadoras les solicitaron a las enfermeras asistenciales información sobre aquellos pacientes hospitalizados en el servicio con padecimientos crónicos. Posteriormente las investigadoras se acercaron a los pacientes y sus familias invitándolos a participar de la investigación y se logró establecer una relación de empatía o rapport(18), que permitió a cada uno de los participantes conocer la investigación y el propósito de la misma, lo que facilitó la participación voluntaria. Dos de las investigadoras con formación en maestría y conocimiento de la investigación cualitativa realizaron 15 entrevistas a 14 participantes, el tiempo empleado para estas fue de 2 horas por participante. Se utilizó un formato con preguntas orientadoras abiertas, con el fin de favorecer una conversación libre y espontánea, Las entrevistas se realizaron en la habitación del paciente, estuvieron presentes el paciente, cuidador informal en algunas ocasiones y dos de las investigadoras. Al ingreso del personal del hospital a la habitación esta se suspendía y continuaba cuando estos se retiraban.

Estas fueron grabadas en medio magnético y transcritas inmediatamente por una estudiante del semillero de investigación, posteriormente revisadas por las investigadoras. Para garantizar la confiabilidad de los datos a cada entrevista se le asignó un código y se cambió el nombre del entrevistado por un seudónimo y los datos que permitieran identificar la institución donde se realizó para mantener la confiabilidad. Durante todo el proceso investigativo se llevó el diario de campo(19) en el que se registraron todos los asuntos de las entrevistas inmediatamente después de realizarlas, para evitar omisiones u olvidos de información que pudieran ser relevantes, igualmente se utilizaron para escribir las observaciones, la observación fue realizada por una de las investigadoras por periodos de 2 horas con una guía al inicio se observó el entorno hospitalario los espacios, los actores, objetos, eventos, la interacción enfermera - paciente - cuidador informal relacionada con las intervenciones para garantizar la comodidad en el entorno hospitalario. El diario de campo aporto a la construcción del texto final. El trabajo de campo tuvo una duración de 6 meses.

El análisis se realizó de forma manual con ayuda de un computador; se tuvo en cuenta las herramientas de la teoría fundamentada(20) para el análisis en el cual se realizó: una codificación abierta en la cual hubo lectura y relectura de los relatos partiendo de la segmentación de ellos sin descontextualizarlos, para lograr el significado en todo su conjunto, para lo cual se siguieron los siguientes pasos: - Recuperar los datos codificados asignándoles un código particular, - Explorar los códigos y las categorías creados a través de una reclasificación asignándoles un nuevo nombre, - y luego a la generalización y teorización; en la codificación axial, se identificaron los conceptos para categorizarlos, relacionando las categorías con sus subcategorías(21), el muestreo teórico(20) permitió hacer más densas las categorías y alcanzar la saturación teórica; es decir, hasta que los datos no aportaron nada nuevo al desarrollo de las propiedades y dimensiones de las categorías que emergieron para dar respuesta al objetivo del estudio(22).

Durante el proceso investigativo se mantuvo el rigor metodológico considerando los criterios de credibilidad, transferibilidad, dependencia, y auditabilidad(23). Igualmente se consideraron los aspectos éticos básicos de beneficencia, no maleficencia, justicia y respeto a la autonomía. Se tuvo en cuenta la resolución 008430 del Ministerio de Salud(24), para la calificación del riesgo, la confidencialidad de la información, la garantía de cuidar la privacidad de los informantes, el respeto a la autonomía al aceptar o no la participación en la investigación, el uso de los datos con fines académicos y la devolución de los resultados a los participantes e instituciones. lo mismo que la Ley 266 de 1996(25) por la cual se reglamentan los principios que rigen la profesión de enfermería y la Ley 911 del 2004 que dispone la responsabilidad deontológica para el ejercicio de la enfermería en Colombia(26). El proyecto se presentó al Comité de Ética de Investigación según Acta 083 del Comité Técnico de Investigaciones de la institución que aprobó la ejecución del proyecto.

## Resultados

Descripción del contexto hospitalario. El estudio se llevó a cabo en un hospital de 4 nivel de complejidad con una amplia oferta de servicios, como consulta general y especializada, ayudas diagnósticas, urgencias, cirugía, hospitalización, unidades de cuidados intensivos y especiales, trasplantes; además, desarrollan investigación e innovación. la institución tiene un modelo de atención centrado en el paciente y la familia.

Los servicios de medicina interna donde se encuentran internados los pacientes con enfermedad crónica están ubicados en el bloque 1 de la institución. Existen unos servicios con características especiales como son el VIP, el servicio de hematología y el de pacientes con aislamiento. Los pacientes entrevistados se encontraban hospitalizados en el 3, 6, 7 y 8 piso. Cada piso cuenta con dos servicios uno en el ala norte y otro en el ala sur. Este tipo de servicios tiene capacidad para 30 pacientes aproximadamente ubicados en habitaciones compartidas y algunos en habitación individual, con una infraestructura y disposición de espacios similar.

En el Diagrama 1 se presentan las características de algunos de los servicios donde se encontraban los participantes según lo observado por las investigadoras. En el turno diurno, el equipo de enfermería estaba conformado por 2 enfermeras, una para la gestión del servicio y otra para las actividades asistenciales específicamente para los procedimientos y las altas, 5 auxiliares de enfermería las cuales se encargaban del confort del paciente y la administración de medicamentos, cada una tenía a su cuidado 6 o 7 pacientes. En el servicio VIP eran 4 auxiliares de enfermería. Un médico general, un médico internista. Además, como personal de apoyo durante el día los servicios cuentan con una secretaria y una persona de oficios generales.

**Diagrama 1:**
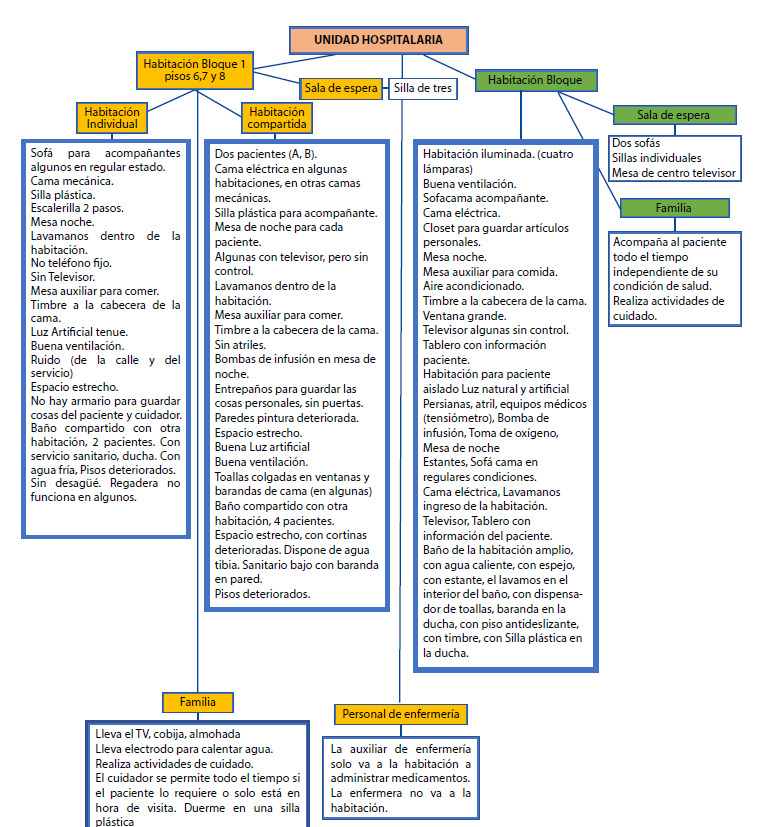
Características de los servicios

Caracterización sociodemográfica. Participaron 14 pacientes (9 mujeres y 5 hombres), edad promedio de 64.7 años, mínima 35 y máxima 88 años. Estado civil: 5 solteros, 3 casados, 1 separado, 2 viudos y 3 en unión libre. En cuanto a la escolaridad: 2 sin formación, 6 primaria incompleta, 1 con bachillerato incompleto, 1 con bachillerato completo y 4 con estudios universitarios. 8 se dedican al hogar, 2 asalariados y 4 pensionados. De los participantes 12, procedentes de Antioquia, 1 de Bolívar y 1 de Choco. 11 Vivian en el área metropolitana de Medellín, 1 en Marinilla y 1 en Choco.

En cuanto al padecimiento, 12 tienen alteración del sistema cardiovascular, 7 del sistema endocrino, 1 del sistema inmunológico, 1 en el sistema respiratorio y 2 de otros. Ninguna de estos es excluyente.

Con relación al tiempo que llevan con el diagnóstico de su enfermedad crónica 5 de los participantes fueron diagnosticados hace menos de 5 años; 3 entre 5 y 10 años; 2 entre 10 y 15 años; 2 hace más de 20 años y sin información fueron 2 participantes.

Los motivos de ingreso al servicio de hospitalización se debieron a la exacerbación o descompensación de su enfermedad crónica, algunos de los diagnósticos fueron: diabetes mellitus tipo 2, Hipertensión arterial, cardiopatía isquémica, EPOC, Cirrosis hepática, dermatomiositis, IAM, Insuficiencia renal crónica, Fibrilación auricular, cáncer.

En cuanto al acompañamiento de un cuidador durante la hospitalización 12 de los participantes cuentan con este apoyo y 2 no tienen cuidador durante la hospitalización. El parentesco que tienen con estos es en su mayoría el de hija(o); 8 participantes, seguido de esposa 2 y otro familiar 2. A continuación, se describen las categorías emergentes:

**Tabla 1:** Categorías emergentes

Categoría	Subcategoría
Interactuando con otros	El compañero del lado La muerte de otro
Interactuando con el equipo de salud	Atributos de la atención; Entre la comodidad e incomodidad con los que atienden
Elementos	Comunicación Información

*Interactuando con otros.* Se relaciona con las interacciones que como pacientes tienen al estar hospitalizados, una de ellas tiene que ver con el compañero del lado, ya que los participantes que pertenecen al Plan de Beneficios en Salud comparten la habitación con otra persona, esta interacción influye en la comodidad; así mismo, la relación con el equipo de salud, de una forma u otra puede tener implicaciones en la comodidad o incomodidad al estar hospitalizado.

*El compañero del lado:* la componen la relación con el otro y la muerte del otro. La relación con el otro está dada por el compañerismo con el paciente del lado, pues hace parte de la comodidad ya que es compañía, al establecer interacciones amistosas, de colaboración y solidaridad como lo expresan:

*“…uno busca la compañía, ¿no? Porque si yo estoy aquí sola y aquí hay otra persona, yo trato de llevarla con esa persona bien, ¿cierto? Porque necesitamos la compañía, porque uno solo y habiendo con quien conversar, sería como extraño, ¿no? ...” E8*

*“…en habitación compartida los pacientes se entretienen jugando cartas con el compañero y conversando con los acompañantes”. Nota de campo*

Pero tener de compañero a un preso, genera incomodidad; por un lado, porque es un ser humano al que no conoce y también por la presencia de la policía:

*“Y en estas partes las cosas no son muy buenas, pero esta uno un poco mejor que arriba, arriba de hecho, ella me estaba contando que por allá tenían unos presos ¡Dígame con una gente sin saber quién es, la policía y todo ahí... ahí no!”. E11*

*La muerte del otro*: en la cultura occidental es un tabú y un asunto que genera miedo y terror como lo dicen los participantes, pues ver morir genera incomodidad.

*“…si muy importante, como, así como anoche se fue una señora de ahí y por la noche falleció un señor y a mí me da terror, yo tenía mucho miedo, cuando llego la señora fue lo más maravilloso para mí que tuviera esa compañía, porque entonces yo ya”. E5*

*Interactuando con el equipo de salud.* Esta categoría está compuesta por las subcategorías:

*Atributos de la atención*; y *Entre la comodidad e incomodidad con los que atienden*.

*Atributos de la atención:* Entre los atributos de la atención que brindan comodidad están la atención oportuna, y el buen trato.

*La atención oportuna:* la refieren los participantes cuando reciben buena atención de las enfermeras, que pasan constantemente por las habitaciones; por lo tanto, no es necesario llamarlas para satisfacer alguna de las necesidades. Sentirse atendido para mejorar el estado de salud cuando se presenta síntomas que son desagradables y el personal de enfermería está presente para ayudarlos hace parte de la comodidad. Como dicen “llegan a tiempo”:

*“pues hace dos madrugadas atrás tuve una crisis respiratoria que empezó muy sencilla, estaba durmiendo y empecé a sentir frio, el frio se convirtió en temblor y el temblor ya se convirtió en asfixia, muchachas más o menos emm manejando tiempos, creo que en cuestión de cinco minutos aquí estaban prácticamente casi todas las muchachas de este sector, pues eran los auxiliares, la jefe de enfermeras y el doctor, o sea todo el equipo, en cinco minutos y desde el momento en que comencé la crisis “*

- ***El buen trato:*** la comodidad también depende del saludo, como dice el manual de Carreño, el saludo es una norma de cortesía y más cuando se trabaja con pacientes en estado de enfermedad y vulnerabilidad. También se relaciona con sentirse auxiliado o ayudado, como también bien tratado, no sentirse discriminado al realizarle cualquier procedimiento, ese trato es parte de sentirse cómodo como lo refieren:

*¨es el trato de las personas que interactúan con uno diario, como son los enfermeras o enfermeras, médicos, bueno es como el personal que está auxiliándolo a uno... los enfermeros, enfermeras y médicos pues me han tratado muy bien… me tratan muy bien¨E7*

El buen trato no solo lo reciben del personal asistencial sino también del personal administrativo y toda la cadena de atención ya que son respetuosos, eficientes, responsables, y cada uno cumple con el rol que le corresponde.

*“porque lo que yo he visto lo que vivido son excelentes, son excelentes aquí desde el más pequeño hasta el más grande uno lo ve que con el respeto, con la eficiencia, con el trabajo, la responsabilidad, el tiempo que estaba aquí no he visto que un superior ha llamado la atención a un subalterno ha llamado la atención en el servicio, cada uno a lo que tiene que hacer”.* E15

Asuntos como estar ahí, la amabilidad y las cualidades personales hacen que la persona se sienta cómoda y así lo refieren de todo el personal.

*“Pues primero porque ella está conmigo y segundo porque todos aquí son muy amables con uno, muy formales, hace que uno se sienta bien… pues personas agradables, que sientan que uno se sienta bien en el ambiente de ellos”. E1*

*“…durante el tiempo de observación el acercamiento del personal de enfermería hacia los pacientes y cuidadores informales, se dio para la administración de medicamentos, el cuidador es quien asiste al paciente en el baño, la alimentación, el acicalamiento”.*

Nota de campo:

*Entre la comodidad e incomodidad con los que atienden*:

*Los que generan comodidad.* Parte de lo que las hace sentir cómodos es la atención que le brindan los médicos, la cual es catalogada como buena porque se sienten bien atendidos, son formales y cubren sus necesidades de atención.

*“los médicos si han estado pendientes, de hecho, me llevaron a hacer ese examen, pero estuve como dos días esperando que me dijeran que era lo que me iban a hacer, hasta que el medico dijo que me tenían que días, yo no sé si fue el martes o miércoles, no recuerdo, me llevaron a hacerme un examen para poderme decir que me tenían que hacer el cateterismo, entonces la doctora, o sea donde me lo hicieron, me dijeron que había salido alterado, que no me podían hacer eso… Después mi hija fue y hablo con el médico y todo, y dijo: “No hay que volverle a hacer el examen, ese se lo hago yo personalmente”, el cardiólogo. Entonces, el me lo hizo ayer de mañana, en la mañanita, a las 6 de la mañana me tenían por allá. Muy formales, muy atentos, muy buena atención allá”. E9*

Pero no todos los médicos comparten los mismos atributos, los catalogan también “más o menos” y “los queridos”; entre más lindos sean más comodidad brindan.

*“de los médicos, porque hay unos que son más o menos, otros muy lindos muy queridos, ay si se siente uno muy cómoda”. E3*

Como el hospital recibe estudiantes del área de la salud se refieren a ellos como personas que los tratan muy bien.

*“pues yo creo que, todo el personal, todo el personal porque vienen estudiantes tratan muy bien a uno, uno se siente bien con ellos, que, si habrá gente que, si trata a uno ahí como a las malitas, pero no, yo no tengo nada que decir de ellos muy bien”. E5*

La visita administrativa a los pacientes la ven como un asunto positivo, porque demuestra interés por las necesidades de los pacientes.

*“muy formal aquí han venido todos los de digamos ¿cómo es la doctora que maneja esto? la gerente ¿cómo es? unos de la universidad cómo será que se llaman, pues toda esa gente ha venido a preguntar todo referente aquí conmigo”. E12*

Los que generan comodidad, tienen atributos como estar pendientes, ser delicados, amables tener buena voluntad, dar ánimo, y tener preparación:

*Las Enfermeras pendientes* están constantemente dándoles vuelta a los pacientes y a su debido tiempo le dan el medicamento que corresponde.

*“…aquí, hay unas que, si están pendientes, si están viniendo, entonces no tiene uno que tocar. Pero hay otras que vienen por allá cuando…Las enfermeras, hay unas que… ayer, por ejemplo, la del turno de ayer, no era muy buena, pero ¡bueno!... Pues se comportan, en que siempre están pendientes de uno, que necesita, le están dando vuelta, le trae la medicina o lo que a uno le toca a su debido tiempo… Por ahí hay una que ella cuando le toca este lado, ella está pendiente todo el tiempo… hay enfermeras, no todas, unas muy especiales, o sea son muy atentas”. E9*

Atributos personales de quien atiende como la amabilidad, la extroversión, el conversar con el paciente y la satisfacción de las necesidades de los pacientes son atributos que generan comodidad.

*¨…no me he encontrado con el primero que sea… que yo pueda decir que es repelente, que es mala clase, ¡No! Gracias a Dios… Pues acá me ha parecido bien, porque yo apenas vine a las doce de la noche, pero si excelente, aquí es bueno. [acompañar] … Pues hasta aquí sí, ellos no me parecen mala clases, aquí está bien… Hasta aquí bien, excelente. Los enfermeros muy queridos y las enfermeras también, muy queridos… También puede ser el buen genio de los auxiliares, de los… pues de las personas que están a nuestro cargo¨. E8*

Igualmente, conocer quien lo atenderá a uno en el turno es de vital importancia para los pacientes y de esto refieren.

*“la comunicación, las auxiliares, o sea, uno dice muy coloquialmente, hay de todo, pero yo podría colocar en un porcentaje, un 90% de muy buena comunicación, no solamente el hecho de que lleguen cuando toman el turno, se le presentan a uno, soy julanita voy a estar todo el día ta ta ta ¿sí? sino que, si voy a tomar una droga, me hablan de la droga si me llegan con el alimento, me explican por qué el alimento, si yo pregunto…” E13*

Las enfermeras que dan ánimo y alientan a los pacientes con sus palabras confortan y brindan comodidad.

*“A ver cómo le explico, ellos me tratan bien, me ven triste las enfermeras me dicen una cosa, pues me dan como ánimos, ellos me tratan muy bien, la droga me la dan muy bien, pues a mí me parece muy bien la atención de acá de ellos, me siento cómoda, sí”. E4*

No solo los atributos personales y humanos son importantes para los pacientes, sino los conocimientos y técnicas son de interés para sentirse cómodo y seguro.

*“…y otra cosa es también ver que las personas están bien preparadas, que sepan hacer bien sus cosas. Ah, ¿qué van a canalizar una vena? Que se vea que, si saben, que no son chúcelo a uno y nada, pues la preparación…” E10*

*Los que generan incomodidad.* Entre ellos están los atributos del personal como lo médicos que no escuchan al paciente:

*“yo hablaba con la doctora, y le decía doctora, con una sola doctora que tuve una diferencia, porque ella pensaba que algunas de las pruebas era que yo no me las quería hacer, por ejemplo, necesitan el cateterismo y entonces eran once horas de inmovilidad de la pierna, entonces le decía, no es que yo no quiera que me hagan la prueba, sino que yo quiero que ustedes me garantizan que en esas once horas voy a poder descansar sobre mi espalda, voy a poder voltear un poquito”. E13*

O los malgeniados, atributo que no genera comodidad en los pacientes

*“Porque hay unos que bendito Dios, son como muy malgeniados o malgeniadas. No le digo pues que entran y uno no se da ni cuenta si entran o no entran porque no saludan y hay muchas que son hasta con las que hacen el aseo”. E3*

A pesar del anhelo por parte de los participantes de sentirse bien tratado, lamentablemente no siempre es así.

*“… mientras que hay otras que no le paran bolas a uno”. E10*

Otro de los nombres con que titulan a las enfermeras, son las que no aparecen a pesar de que son llamadas para satisfacer alguna de las necesidades.

*“pero hay otras que a veces uno se cansa de llamar y no aparecen… para uno pues que necesita una atención de ellos, pues no debiera de ser así”. E9*

Son las enfermeras que tardan mucho al llamado, las que aparecen de vez en cuando, dejan esperando a los pacientes y ellos se sienten desatendidos. Muchas veces los pacientes se cansan de tocar el timbre que es la única comunicación que pueden tener con el puesto de enfermería.

*“Hay otras que, por allá, de repente vienen … tardan para venir y eso cuando uno las llama… vienen de repente… si, necesito preguntar algo, que me den algo y eso. Entonces uno llama, llama y llama y al tiempo es que vienen... hay otras que uno se cansa de tocar el timbre y no aparecen. Entonces esas son las cosas, no solo a mí, hay muchos que no les gusta eso”. E9*

El lenguaje no verbal también es importante al hablar de comodidad y habla del buen trato.

*“porque no es necesario palabras para uno sentirse mal…por el lenguaje, como usan los utensilios, como lo manipulan a uno, ese es un lenguaje que lo puede hacer sentir o muy bien o muy mal”. E7*

*Elementos:* *La comunicación con el equipo de salud* es a través del timbre, pues en las habitaciones no hay teléfono, el timbre supuestamente es utilizado cuando requieren ayuda.

*“hay timbre, aquí teléfono no hay”. E9*

*“Pues de lo que hay, pues que hay unos elementos ahí que le ayudan en cualquier momento los usan para lo que están tratando a uno, que hay un botón para llamar a la enfermería si uno requiere”. E7*

La comunicación que tengan con el celular depende de la señal, por ejemplo, en algunos servicios hay señal de celular en otros no hay señal.

*“esta semana he estado muy incomunicada, porque como estuve toda la semana abajo, entonces no…hoy si, aquí hay mejor señal, entonces como el celular es un viejito ahí pequeñito, último modelo, entonces no le entra mucho la señal”. E8*

Como las habitaciones no tienen teléfono la comunicación con la familia ha de ser por el teléfono celular, que a pesar de la distancia acerca a la familia y sienten que a través de él hay compañía.

*“con mi familia, no es que aquí no hay teléfono, sino que tiene uno que tener un celular… Claro, ¿porque si uno no tiene un celular, como llama?... Claro, estar en contacto, porque si a uno le llega a pasar algo, pues el que está al lado, agarra y llama, pero si uno no tiene un teléfono, no se puede comunicar, porque a veces que no todos pueden estar al pie de uno, sino a veces tiene que quedarse uno solo, porque pues ellos todos trabajan y no pueden estar a la vez pendientes de uno, entonces con un teléfono uno agarro y marco y ya, pero si uno no tiene celular…” E9*

*La información.* Para los pacientes es importante que el personal de salud se ponga de acuerdo en el momento de dar la información.

*“Como segura de lo que me están haciendo y que los médicos y los mismos enfermeros se pongan de acuerdo, que lo que le van a decir a uno sea lo mismo, porque a veces dicen unas cosas uno, el otro dice otras, el otro le agrava a uno el momento, el otro se lo pone más relajado”. E10*

La falta de claridad en el diagnóstico genera incomodidad

*“al internista le dije yo molestando, yo le voy a poner el rompecabezas, le dije yo le voy a poner el rompecabezas porque usted tiene que organizar todo esto, la dermatomiositis, lo de la calcinosis, lo del azúcar, lo de que fue o no fue infarto ¿sí?, yo ya tengo unas drogas de base que son estas estas y estas, ya lo nuevo que hay que tomar, yo por ejemplo tengo que saber si en este momento toda esta debilidad, toda la debilidad que estoy sintiendo, hasta qué punto es por el infarto y por el estado en que estoy y qué otra parte de la debilidad tiene que ver con la dermatomiositis”. E13*

## Discusión

En este apartado se discutirán los resultados de la investigación con la literatura relacionada. El estudio nos aportó comprensión de diferentes asuntos que generan comodidad e incomodidad entre los participantes, respecto al compañero de lado que reporta nuestro estudio, la convivencia o el compartir la habitación con otros pacientes es un asunto que tiene doble arista como lo expresa la tesis de Belmar-Estrada P y et al., pues la convivencia diaria con un paciente crítico genera sentimientos negativos, como “frustración, pena, intranquilidad e impotencia, afectando el estado anímico”. Pero también surgen sentimientos positivos destacando la empatía, protección y ayuda que nacen entre ellos durante la convivencia(27). Así mismo, compartir la habitación con un preso genera inseguridad, como lo manifiestan Pallares Martínez E et al., al referir que la convivencia con el interno del sistema penitenciario genera situaciones que pueden comprometer la seguridad tanto del compañero del lado, como del personal de enfermería y otros que desempeña su labor en la sala(28). Tanto el personal del hospital, como el de la prisión deben equilibrar la privacidad, confidencialidad y autonomía del paciente con la necesidad de garantizar la seguridad de los otros pacientes como del personal del hospital. Esta tensión puede generar conflictos entre los cuidadores familiares, formales y pacientes. Igualmente, la presencia de oficiales armados, pueden generar temor en los pacientes y acompañantes(29).

La muerte del otro es un fenómeno complejo, ambiguo y desconocido. Morir es un proceso individual, y un acontecimiento que afecta a los allegados de quien muere. De allí que las actitudes y comportamientos que las personas adoptan ante la muerte sean el resultado de características y circunstancias individuales, por un lado, y del concepto y sentido de la muerte imperante en la sociedad, ya que la muerte como producto de la sociedad occidental genera actitudes y comportamientos que son aprendidos culturalmente; ya que esta moldea las experiencias de pérdida y los rituales que la rodean. La mayoría mueren en el hospital(30) y afecta a la persona de al lado, que sufren por la pérdida, ya antes de que se produzca, cuando ésta no es de manera repentina(31).

Interactuando con el equipo de salud. Como lo presenta nuestro estudio los resultados son compatibles con el estudio de Homar Amengual C, et al.,(32) que se refieren a la empatía, la competencia profesional y el uso de un lenguaje adecuado como las cualidades más nombradas. La actitud de los trabajadores de salud, médicos, administradores, enfermeras y otros trabajadores de la salud es motivo de preocupación. Las buenas relaciones de persona a persona entre los trabajadores de la salud y los pacientes en la entrega de servicios de salud dan forma a la percepción positiva del paciente y contribuirá a mejorar la imagen del hospital(33).

Según el estudio de Otani K, et al(34)., los atributos de la atención de la salud, como la atención de enfermería, del médico y las comodidades de la habitación, están relacionadas con la satisfacción general. Este estudio encontró que algunos atributos son más influyentes que otros en la satisfacción general como la atención de enfermería es más influyente en un entorno hospitalario y la atención médica es más influyente en un entorno ambulatorio.

Este autor describe 4 asuntos (enfermería, médico, personal y habitación) relacionados positi- vamente con la calificación general. Entre esos 4 atributos, el cuidado de enfermería muestra el mayor coeficiente. Este resultado es consistente con otros estudios ya que las características del hospital influyen en la calificación general del hospital a través del médico, el personal y los atributos de la habitación(34).

Como lo expresa el presente estudio la comunicación verbal y no verbal son vitales; la verbal durante el padecimiento de una enfermedad puede ser difícil, por lo que la comunicación no verbal como la postura, la expresión facial, la mirada y los gestos cobra mucha importancia; por tanto, el profesional de enfermería debe tener presente lo que expresa su corporalidad y la del paciente en toda su complejidad, sin que lo manifieste verbalmente(35). Así la comunicación verbal y no verbal están presentes en las interacciones personales del cuidado y están sujetos a una serie de condicionantes personales y ambientales que pueden influir en dicha relación(35).

Las comunicaciones eficientes entre el paciente y el equipo de salud son importantes en la relación y las quejas de los pacientes no se relacionan con las habilidades del médico, sino con una comunicación ineficaz que conduce a malentendidos y disminución de la satisfacción del servicio prestado. Muy a menudo, los pacientes se quejan de que los médicos no los escuchan(36).

Los médicos ignoran, aspectos interpersonales como el cuidado, la apreciación y la empatía. Los resultados destacan, por lo tanto, la brecha en la forma en que los pacientes y los médicos experimentan y evalúan la confianza en su relación de atención. Si bien nuestra investigación muestra que los pacientes enfatizan la importancia de sus sentimientos y emociones, los médicos creen pragmáticamente que su desempeño laboral es el indicador más importante para confiar en la relación.

Como lo informa nuestro estudio, el conocer la enfermera que está a su cargo es parte de la comodidad como lo relacionan los resultados del meta-análisis de Henok Mulugeta et al., que relatan que los pacientes que tenían una enfermera a cargo de su atención tenían más posibilidades de estar satisfechos con la atención de enfermería en comparación con aquellos pacientes sin la enfermera asignada a cargo de su atención ***,*** así la disponibilidad de una enfermera asignada a cargo, influyeron en la satisfacción de los pacientes con los cuidados de enfermería(37).

El estudio Ramírez et al., refiere que la comunicación humana entre enfermera y paciente requiere tiempo, e incluye información, comunicación, comprensión y trato digno; sin embargo, para algunos profesionales su desempeño laboral podría significar la realización de procedimientos, sin importar lo que siente o necesita el paciente. Se señala que la enfermera en ocasiones emplea el lenguaje verbal técnico, establece contactos físicos breves y no planificados basados en el cuidado físico y tecnológico sin considerar las emociones(35), lo que contribuye a la incomodidad del paciente como lo expresaron en el estudio.

Las actitudes empáticas sustentadas en sentimiento de comprensión entre dos personas, les permite interactuar mediante acciones individuales con un objetivo común(36).

Para hacerle frente a los hallazgos del estudio, Jean Watson(38), en 2007, creó el *Watson Caring Science Institute* (WCSI), organización internacional que promueve filosofías, teorías, prácticas del cuidado humano en la atención en salud. Además, se refiere al ser humano como un ser sintiente y como tal en la interacción personal con el equipo de salud percibe sensaciones de confort o disconfort, en asuntos relacionados con la comunicación, la información, la sensibilidad personal, entre muchos otros(39); por tanto, se hace necesario el cultivo de la sensibilidad hacia los otros(39).

Así mismo, la ciencia del cuidado es un campo de estudio filosófico-ético-epistémico en evolución que se basa en la disciplina de la enfermería y en campos relacionados que tiene al amor como punto de partida para practicar el cuidado humano que tiene explicitó la conexión con las artes y las humanidades(39). Así esta teoría se relaciona con los asuntos subjetivos que ha de tener el equipo de salud al interactuar con el paciente para brindar comodidad.

Parte de ese cuidado humano está en la información, para los pacientes es importante la información relativa a su enfermedad, el curso de esta, y los resultados del tratamiento, y sus efectos secundarios y consejos sobre lo que pueden hacer por sí mismos(36). Consideran que la tecnología es fundamental, sin embargo, lo más importante del cuidado de enfermería son los aspectos humanos como la compasión, los estímulos y la atención, así como brindar consuelo para aliviar el miedo y la inseguridad. Cuando la enfermera es amable y empática se reducen los sentimientos de miedo(35). Así mismo, la comunicación es una interacción interpersonal compleja que requiere una comprensión del estado emocional de cada parte(36)

En la actualidad, las ciencias de la salud están evolucionando rápidamente, centrándose en la tecnología y las ganancias. Como resultado, los sistemas de salud, los hospitales y las aseguradoras han trasladado el foco a la satisfacción del paciente(36).

Los atributos del personal hacen parte de la calidad del servicio con un valor alto, seguido de la comunicación(33) y todos estos asuntos apuntan a la satisfacción de los pacientes, el cual se considera un indicador de gestión. A partir de este indicador se puede obtener la opinión acerca de los aspectos de la estructura (comodidades, instalaciones físicas, organización), el proceso (procedimientos y acciones realizadas durante la hospitalización) y el resultado (cambios en el estado de salud y la percepción general de la atención recibida)(40).

Esta investigación aporta a la práctica de enfermería en el sentido que esta ha de ser más humana y consciente de las necesidades del otro. Por tanto, desde la enfermería es necesario crear una cultura del cuidado humano que engloba todos los aspectos que consideran los participantes como asuntos de la comodidad. Así mismo, es necesario continuar la investigación en todas las dimensiones del cuidado en especial desde la visión de los pacientes y que los resultados si sean integrados a la praxis de enfermería.

Entre las limitaciones del estudio se relacionan con la generalización pues los hallazgos y su análisis es producto de la recolección directa con los participantes del proyecto en un contexto local pudiendo no reflejar la realidad de otros en otros contextos.

## Conclusión

Este estudio encontró que la relación con los otros influye positiva o negativamente en la comodidad de los pacientes durante la hospitalización. Esta relación está dada por la interacción con el paciente del lado y con el equipo de salud del cual sus actitudes frente al paciente y sus atributos personales cobran una importancia inconmensurable.

Parte de la comodidad está dada por la información que requiere el paciente de su estado y evolución de salud como de los efectos de su tratamiento.

Los elementos de comunicación se hacen indispensables por un lado para comunicarle al personal de alguna necesidad por medio del timbre o para estar comunicado con la familia y sentirla cerca.

Algunos de los aspectos que causan incomodidad son: el no ser escuchados por el personal de salud, la actitud negativa del personal de enfermería, el no atender a su llamado oportunamente y ver morir al compañero del lado.
